# Tumor-Intrinsic PD-L1 Signaling in Cancer Initiation, Development and Treatment: Beyond Immune Evasion

**DOI:** 10.3389/fonc.2018.00386

**Published:** 2018-09-19

**Authors:** Peixin Dong, Ying Xiong, Junming Yue, Sharon J. B. Hanley, Hidemichi Watari

**Affiliations:** ^1^Department of Obstetrics and Gynecology, Hokkaido University School of Medicine, Hokkaido University, Sapporo, Japan; ^2^Department of Gynecology, State Key Laboratory of Oncology in South China, Sun Yat-Sen University Cancer Center, Guangzhou, China; ^3^Department of Pathology and Laboratory Medicine, University of Tennessee Health Science Center, Memphis, TN, United States; ^4^Center for Cancer Research, University of Tennessee Health Science Center, Memphis, TN, United States

**Keywords:** PD-L1, CD274, metastasis, EMT, cancer stem cells, microRNA

## Abstract

Although the role of PD-L1 in suppressing the anti-tumor immune response is extensively documented, recent discoveries indicate a distinct tumor-intrinsic role for PD-L1 in modulating epithelial-to-mesenchymal transition (EMT), cancer stem cell (CSC)-like phenotype, metastasis and resistance to therapy. In this review, we will focus on the newly discovered functions of PD-L1 in the regulation of cancer development, describe underlying molecular mechanisms responsible for PD-L1 upregulation and discuss current insights into novel components of PD-L1 signaling. Furthermore, we summarize our current understanding of the link between PD-L1 signaling and the EMT program as well as the CSC state. Tumor cell-intrinsic PD-L1 clearly contributes to cancer stemness, EMT, tumor invasion and chemoresistance in multiple tumor types. Conversely, activation of OCT4 signaling and upregulation of EMT inducer ZEB1 induce PD-L1 expression in cancer cells, thereby suggesting a possible immune evasion mechanism employed by cancer stem cells during metastasis. Our meta-analysis demonstrated that *PD-L1* is co-amplified along with *MYC, SOX2, N-cadherin* and *SNAI1* in the TCGA endometrial and ovarian cancer datasets. Further identification of immune-independent PD-L1 functions and characterization of crucial signaling events upstream or downstream of PD-L1 in diverse cancer types and specific cancer subtypes, would provide additional targets and new therapeutic approaches.

## Introduction

In cancer, the epithelial-to-mesenchymal transition (EMT) is a phenotypic process that promotes the acquisition of a mesenchymal features of epithelial tumor cells, reduces cell polarity and cell-cell adhesion, and enables them to migrate and invade more efficiently, by switching off the expression of epithelial markers, such as E-cadherin, and turning on mesenchymal markers, including N-cadherin and Vimentin (1, 2). Epithelial tumor cells undergoing EMT are shown to contribute to tumorigenesis, invasion, metastasis, and resistance to chemotherapy, radiation and small-molecule-targeted therapy (3).

Cancer stem cells (CSCs) represent a fraction of undifferentiated cancer cells that are the seeds of tumor recurrence, have the ability to self-renew and exhibit significant resistance to conventional chemo- and radiotherapy (4). Emerging evidence has revealed an association between EMT and the acquisition of CSC-like properties (5). The induction of EMT program is a critical regulator of the CSC phenotype (6, 7). On the other hand, tumors cells that exhibit the CSC phenotype also express genes associated with the EMT features and show enhanced metastatic ability, thus representing a novel mechanism contributing to cancer metastasis (8).

The mutual interactions between tumor cells and the tumor microenvironment are essential for tumorigenesis, tumor progression, metastasis and resistance to drug therapy (9). Tumor microenvironment consists of extracellular matrix and diverse cell populations such as T cells, NK cells, macrophages, dendritic cells, fibroblasts, and endothelial cells (10). Progression of cancer to an advanced or metastatic disease usually suggests a failure or insufficiency of the ongoing immune response. Tumors not only effectively escape immune recognition, they also actively inhibit T-cell-mediated normal anti-tumor activity to promote further tumor growth and metastasis by modulating immune checkpoints, which mediate immune tolerance and inhibit the anti-tumor immune response (11). Multiple checkpoint molecules, such as PD-1/PD-L1, CTLA4, BTLA, B7H3, B7H4, HHLA2, IDO1, Tim-3, CD28, CD40, CD47, CD70, CD137, VISTA, LAG-3, and TIGIT, have been reported (11). Among them, B7H3 has been identified as a critical promoter of tumor cell proliferation, migration, invasion, EMT, cancer stemness, and drug resistance (12).

PD-L1 (also known as CD274 or B7H1) is expressed in tumor cells and plays a crucial role in tumor immune escape and the formation of a permissive immune microenvironment, through at least three mechanisms: (i) tolerizing or anergizing tumor-reactive T cells by binding to its receptor PD-1; (ii) rendering tumor cells resistant to CD8^+^ T cell and Fas ligand-mediated lysis; and (iii) tolerizing T cells by reverse signaling through T cell-expressed CD80 (13, 14). In addition, PD-L1 is also expressed by tumor-associated myeloid-derived suppressor cells and macrophages, which are the major factors responsible for tumor-associated immune deficiencies (15).

Although PD-L1 is widely implicated in tumor immune evasion, the tumor-intrinsic roles of PD-L1 and the mechanisms by which PD-L1 regulates EMT, the acquisition of tumor-initiating potential and resistance to anti-tumor drugs, as well as the ability to disseminate and metastasize in human cancers are currently less well defined. As will be discussed in more detail below, the identification of tumor-intrinsic PD-L1 signaling may provide critical targets for the development of cancer therapies.

## PD-L1 dysregulation and prognosis in cancer

An increasing number of studies suggested that PD-L1 is highly expressed in solid tumors, including colorectal cancer (16), lung cancer (17), pancreatic carcinoma (18), hepatocellular carcinoma (19), gastric cancer (20), ovarian cancer (21), endometrial cancer (22, 23), and cervical cancer (24, 25). High expression of PD-L1 was associated with significantly worse overall survival in cervical cancer (25), non-small cell lung cancer (26), gastric cancer (27), esophageal cancer (28), glioma (29), ovarian cancer (30), and other cancers (31). However, the prognostic value of PD-L1 for certain types of cancer is still controversial. Some studies reported that high PD-L1 could predict favorable prognosis (32, 33). In cervical cancer, squamous cell carcinomas tended to express more PD-L1 than adenocarcinomas (34). The possible reasons for these inconsistent results might include cancer type (or subtypes), tumor heterogeneity, sample size, clinical stage, different intervention, the time point of PD-L1 measurement as well as the different methodology used in research (such as detection methods and procedures).

## Mechanisms of PD-L1 activation in cancer

**Figure 1:**
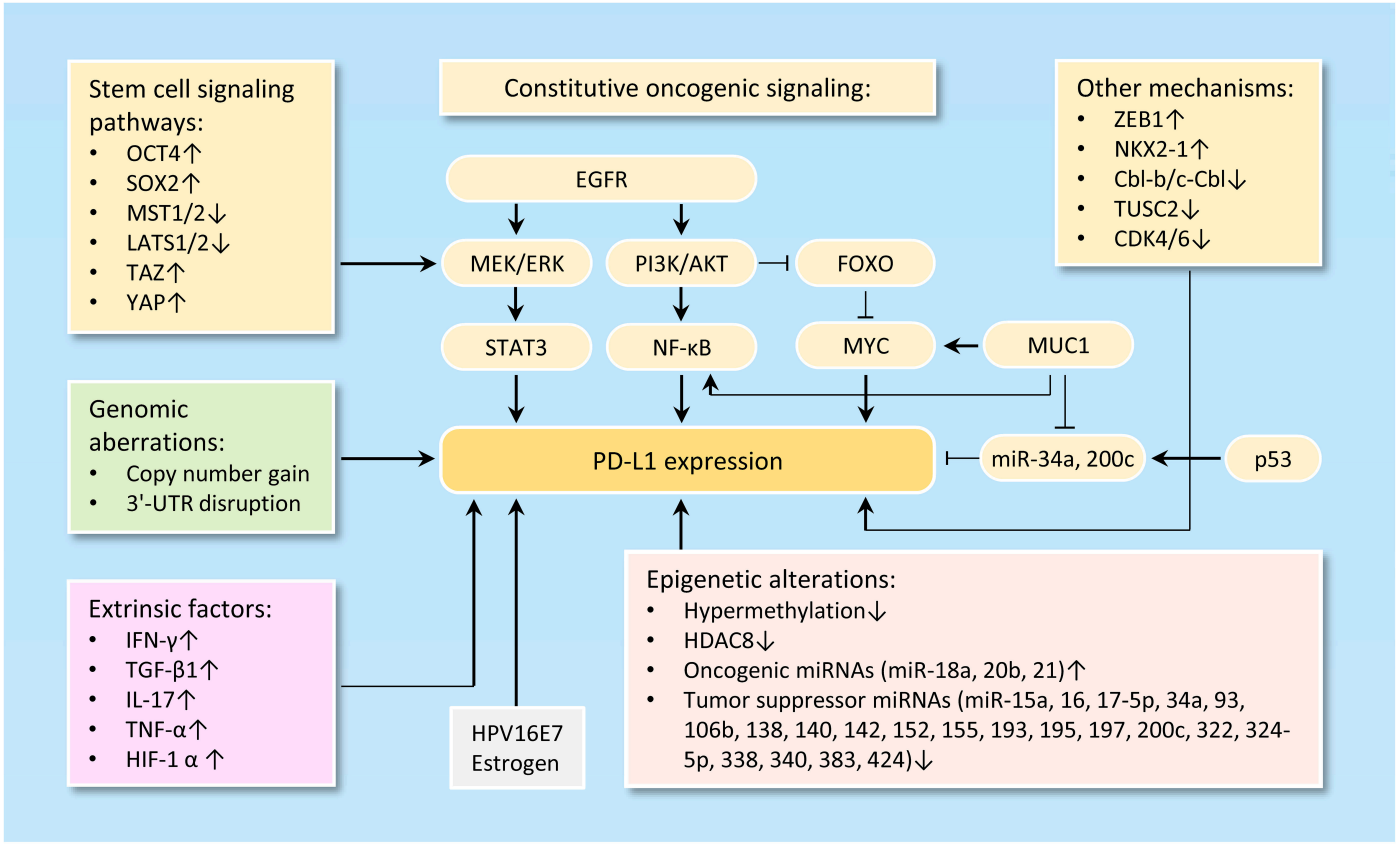
Mechanisms of PD-L1 activation in cancer. The diagram illustrates the diverse mechanisms of PD-L1 activation in cancer, including genetic alterations to *PD-L1* (such as gene amplification, 3'-UTR disruption, or dysregulated transcription) and a wide range of epigenetic mechanisms (including upregulation of oncogenic microRNAs, downregulation of tumor suppressor microRNAs, aberrant DNA methylation and histone modifications).

The tumor-intrinsic PD-L1 signaling pathway is inappropriately activated in many cancers. Mechanisms underlying aberrant PD-L1 activation mainly include genomic alterations (including copy number amplification and 3'-UTR disruption), constitutive oncogenic signaling activation, extrinsic factors and epigenetic mechanisms, such as upregulation of oncogenic microRNAs (miRNAs), downregulation of tumor suppressor miRNAs, aberrant DNA methylation, and histone modifications (Figure 1).

### Copy number gain and 3′-UTR disruption

**Figure 2:**
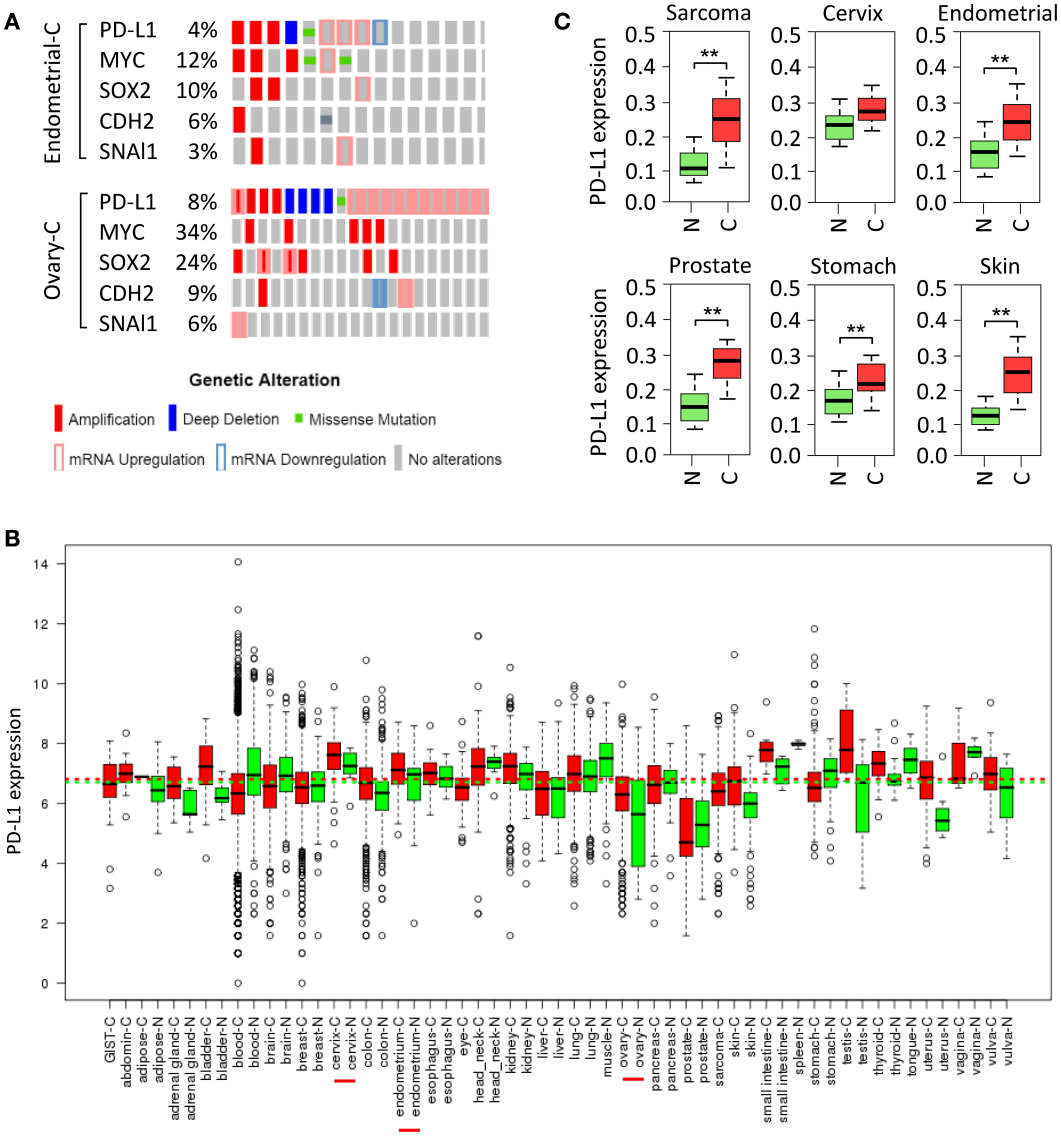
Amplification and upregulation of PD-L1 and genes co-amplified with PD-L1 in TCGA data. **(A)** The Cancer Genome Atlas (TCGA) datasets in the cBioPortal database (www.cbioportal.org) was used to investigate molecular alterations (RNA expression, copy number variation, and mutation). Shown are OncoPrint outputs where each bar represents a tumor that was found to contain an alteration (amplification, deletion, mutation, upregulation, and downregulation, as indicated) in *PD-L1, MYC, SOX2, N-cadherin* (*CDH2*), and *SNAI1* gene in samples of endometrial cancer (upper panel) and ovarian cancer (lower panel) based on TCGA data. **(B)** *PD-L1* mRNA expression pattern was analyzed in a panel of cancer (red) vs. normal (green) tissues from the GENT database. **(C)** *PD-L1* expression pattern was determined in multiple cancer microarray datasets available in the MethHC database. N, normal; C, cancer. **P < 0.005.

Small-cell lung cancer (35), squamous cell carcinoma of the oral cavity (36), cervical cancer (37), ovarian cancer (38), breast cancer (39), melanoma, bladder cancer, head and neck cancer, soft tissue sarcoma and prostate cancer (40) exhibit increased copy number of chromosome 9p24, on which CD274 resides. Here, we investigated the frequency of elevated PD-L1 in ovarian cancer and endometrial cancer in The Cancer Genome Atlas (TCGA) data portal. Analysis of TCGA data by cBioPortal (41) demonstrated that overall, PD-L1 was highly expressed in these two cancers, mainly including gene amplification and mRNA up-regulation (Figure 2A). Moreover, analyses of U133A and U133Plus2 datasets in the GENT (gene expression across normal and tumor tissue) database (42) revealed that *PD-L1* was highly overexpressed in many tumor tissues (Figure 2B). Furthermore, analysis of the TCGA dataset was performed by using the MethHC browser (43). *PD-L1* mRNA expression was consistently upregulated across various cancers (Figure 2C). In addition, disruption of the 3' region of the *PD-L1* increases mRNA stability, leading to a marked elevation of aberrant *PD-L1* transcripts in multiple cancers (44).

### Constitutive oncogenic signaling activation

Loss of PTEN expression, activation of PI3K/AKT pathway, activation of RAS/MAPK pathway, inhibition of p53 signaling, upregulation of reprogramming factors (Oct4, Sox2, and c-Myc) and upregulation of ZEB1 (an inducer of EMT) are clearly linked to the activation of PD-L1 signaling pathway (45, 46) (Figure 1).

PD-L1 expression could be regulated via the PI3K/AKT and/or RAS/MAPK pathways in different tumor cell types (47–49). PD-L1 expression is suppressed by the tumor suppressor gene PTEN. Deletion of PTEN gene results in elevated PD-L1 expression at the translational level by activating the PI3K/AKT signal pathway (50, 51). FOXOs inhibit the expression of PD-L1 through repressing Myc or Wnt/β-catenin signaling pathways in tumor cells (52). MUC1 elevates *PD-L1* transcription by recruitment of MYC and NF-κB (a downstream effector of PI3K/AKT pathway (53) to the *PD-L1* promoter in breast cancer (54). Also, MUC1 was shown to increase PD-L1 levels via downregulation of miR-34a and miR-200c, two direct suppressors of PD-L1 (55–57).

Abnormal activation of stem cell signaling pathways has been implicated in the regulation of PD-L1. OCT4 is a key regulatory gene that maintains the self-renewal properties of CSC and promotes tumorigenesis of cervical cancer cells by miR-125b/BAK1pathway (58). We recently reported that, OCT4 promotes cervical cancer invasion and proliferation by enhancing PD-L1 expression through a miR-18a-dependent mechanism, by which miR-18a upregulates PD-L1 by targeting *PTEN, WNK2* and *SOX6* to activate the PI3K/AKT, MEK/ERK and Wnt/β-catenin pathways and inhibit the p53 pathway (25). In addition, SOX2, a transcription factor that controls tumor initiation and cancer stem-cell functions, can directly bind to the *PD-L1* promoter and transactive its expression, contributing to the increased proliferation of hepatocellular carcinoma cells (59). The upstream kinases of the Hippo pathway MST1/2 and LATS1/2 suppress PD-L1 expression, while TAZ and YAP enhance PD-L1 levels in breast and lung cancer cells (60).

Tumor cells undergoing EMT are shown to share a variety of capabilities with experimentally defined CSC (61). In lung cancer, PD-L1 expression was significantly higher in patients with EMT phenotypes (such as increased SNAI1 and Vimentin expression) compared with those with epithelial phenotypes (62). siRNA-mediated ZEB1 knockdown suppressed PD-L1 expression but promoted E-cadherin expression in esophageal squamous cell carcinoma (63). In agreement with these reports, cBioportal analysis of data on somatic copy number variation and mRNA level using TCGA endometrial and ovarian cancer dataset demonstrated that *PD-L1* is indeed co-amplified along with *MYC, SOX2, N-cadherin* and *SNAI1* in both cancer types (Figure 2A).

Another study reported that transcription factor NKX2-1 bound to the locus of *PD-L1* and induced its expression in mucinous lung cancer cells (64). In non-small cell lung cancer cells, the ubiquitin ligases Cbl-b and c-Cbl inhibit PD-L1 expression by inactivating STAT, AKT, and ERK signaling (65), and overexpression of tumor suppressor gene TUSC2 downregulated PD-L1 expression (66). CDK4 and CDK6 kinase destabilize PD-L1 protein via cullin 3–SPOP, leading to the downregulation of PD-L1 in cancer cells (67).

### Regulation of PD-L1 expression by epigenetic mechanisms

The expression of cancer-associated genes can occur by epigenetic mechanisms, including DNA methylation (68), histone modification (69), chromatin remodeling, and non-coding RNAs (70). The anti-PD-1 therapy could induce PD-L1 promoter methylation and decrease PD-L1 levels in patients with non-small cell lung cancer (71). The class I histone deacetylase HDAC8 acts as an epigenetic inhibitor of PD-L1 expression in melanoma cells via modulating HOXA5 and STAT3 (72). Numerous miRNAs, including miR-15a/miR-16 (73), miR-17-5p (74), miR-93/106b (75), miR-138-5p (76), miR-140/miR-142/miR-340/miR-383 (25), miR-152 (77), miR-155 (78), miR-193 (73), miR-195 (73), miR-324-5p/miR-338 (79) and miR-322/miR-424 (80), have been shown to directly target and inhibit PD-L1 expression in tumor cells. In chemo-resistant non-small-cell lung cancer cells, miR-197 indirectly inhibits PD-L1 expression by regulating the CKS1B/STAT3 axis (81). On the other hand, oncogenic miR-20b and miR-21 inhibited PTEN expression, resulting in PD-L1 overexpression in colorectal cancer (82). Our recent data established that an oncogenic OCT4-miR-18a pathway serves as the key upstream activator of PD-L1 in cervical cancer (27).

### Extrinsic factors influencing the expression of PD-L1

The main regulators of PD-L1 are the interferon-γ (83), inflammatory cytokines such as IL-17 (84) and TNF-α (84), TGF-β1 (85), and HIF-1α (86). Of note, overexpressing HPV16E7 oncoprotein increased PD-L1 protein expression, and knockdown of HPV16E7 resulted in a reduction in PD-L1 protein expression in cancer cells (87). Consistent with this data, PD-L1 protein expression was significantly higher in the normal cervical tissues with HPV infection than those normal cervical tissues without HPV infection (53). Estrogen is a well-known oncogenic driver of endometrial and breast cancer, and it upregulates PD-L1 protein expression in ERα-positive endometrial and breast cancer cells (88).

## The role of PD-L1 in stimulating or inhibiting cancer

**Figure 3:**
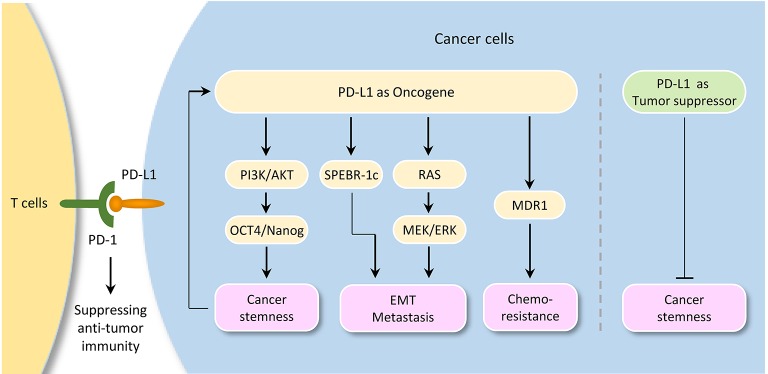
Tumor-intrinsic PD-L1 signaling in cancer initiation and development. The diagram illustrates signaling events downstream of PD-L1 activation in cancer. Although PD-L1 could serve as a tumor suppressor by inhibiting cancer stem cell properties in cholangiocarcinoma, it plays a pivotal role in promoting cancer stemness, EMT, tumor invasion, and chemoresistance in several tumor types. Importantly, activation of OCT4 signaling induces PD-L1 expression in cancer cells, thereby suggesting a possible immune evasion mechanism employed by cancer stem cells during metastasis.

A tumor-intrinsic role for PD-L1 in promoting cancer initiation, metastasis, development, and resistance to therapy is emerging (Figure 3). For instance, knockdown of PD-L1 expression in gastric cancer cells could significantly suppress cell proliferation, migration and invasion (89). Also, knockout of PD-L1 expression by CRISPR/Cas9 inhibits the spheroid formation of osteosarcoma cells (90). PD-L1 was shown to promote EMT in esophageal cancer (91). Knockdown of PD-L1 expression significantly suppressed tumor growth in nude mice in gastric cancer (92) and cervical cancer model (27).

Interestingly, a link between PD-L1 expression and EMT/CSC-like phenotypes has been reported. For example, bladder cancer cells with surface expression of PD-L1 exhibited signatures of immune evasion as well as increased stemness (93). PD-L1 has been shown to be preferentially expressed on CD44^high^ CSCs in lung cancer cells (94). Selective expression of PD-L1 was observed on CD44^+^ head and neck tumor cells compared with CD44^−^ tumor cells (95). CD133^+^/PD-L1^+^ colorectal CSC cells showed the characteristic of EMT (96). Tumor cell-intrinsic PD-L1 promotes tumor-initiating cell generation in melanoma and ovarian cancer (97). Similarly, PD-L1 promotes OCT4 and Nanog expression in breast CSCs through the activation of PI3K/AKT pathway (98).

Moreover, PD-L1 overexpression promotes EMT and invasion in glioblastoma multiforme via RAS/ERK/EMT activation (99). RNA-sequencing analysis of glioblastoma multiforme revealed that PD-L1 significantly altered the expression of genes, which were enriched in cell growth/migration/invasion pathways (99). PD-L1 induced EMT via activating SREBP-1c in renal cell carcinoma (100). CRISPR/Cas9 system-mediated *PD-L1* disruption increased drug sensitivities for doxorubicin and paclitaxel (90). The interaction of PD-L1 with PD-1 induced phosphorylation of AKT and ERK, resulting in the activation of PI3K/AKT and MAPK/ERK pathways and increased MDR1 expression in breast cancer cells (101).

However, depletion of PD-L1 expression by shRNA in cholangiocarcinoma cells enhances their tumorigenicity and increases ALDH activity, and patients with lower PD-L1 expression shows poorer prognosis when compared with those with higher PD-L1 expression (102), indicating that PD-L1 may also have anti-tumor effects by inhibiting cancer stemness under certain circumstances.

## Conclusions

It is becoming clear that, although PD-L1 could serve as a tumor suppressor by inhibiting cancer stem cell properties in cholangiocarcinoma, tumor cell-intrinsic PD-L1 plays a pivotal role in promoting cancer stemness, EMT, tumor invasion, and chemoresistance in several tumor types. Importantly, activation of OCT4 signaling and upregulation of EMT inducer ZEB1 induce PD-L1 expression in cancer cells, thereby suggesting a possible immune evasion mechanism employed by cancer stem cells during metastasis. The continued characterization of immune-independent PD-L1 functions and identification of crucial signaling events upstream or downstream of PD-L1 in diverse cancer types (or specific cancer subtypes), would provide additional targets and new therapeutic approaches.

## Author contributions

PD and HW provided direction. PD, YX, and HW wrote the manuscript. JY and SH made significant revisions to the manuscript. All authors read and approved the final manuscript.

### Conflict of interest statement

The authors declare that the research was conducted in the absence of any commercial or financial relationships that could be construed as a potential conflict of interest.

## References

[1] Shibue T Weinberg RA. EMT, CSCs, and drug resistance: the mechanistic link and clinical implications. Nat Rev Clin Oncol. (2017) 14:611–29. 10.1038/nrclinonc.2017.44 28397828 PMC5720366

[2] Dong P Konno Y Watari H Hosaka M Noguchi M Sakuragi N. The impact of microRNA-mediated PI3K/AKT signaling on epithelial-mesenchymal transition and cancer stemness in endometrial cancer. J Transl Med. (2014) 12:231. 10.1186/s12967-014-0231-0 25141911 PMC4145234

[3] Nieto MA Huang RY Jackson RA Thiery JP. EMT: 2016. Cell (2016) 166:21–45. 10.1016/j.cell.2016.06.028 27368099

[4] Ayob AZ Ramasamy TS. Cancer stem cells as key drivers of tumour progression. J Biomed Sci (2018) 25:20. 10.1186/s12929-018-0426-4 29506506 PMC5838954

[5] Mani SA Guo W Liao MJ Eaton EN Ayyanan A Zhou AY. The epithelial-mesenchymal transition generates cells with properties of stem cells. Cell (2008) 133:704–15. 10.1016/j.cell.2008.03.027 18485877 PMC2728032

[6] Ungefroren H Sebens S Seidl D Lehnert H Hass R. Interaction of tumor cells with the microenvironment. Cell Commun Signal (2011) 9:18. 10.1186/1478-811X-9-18 21914164 PMC3180438

[7] Renner K Singer K Koehl GE Geissler EK Peter K Siska PJ. Metabolic hallmarks of tumor and immune cells in the tumor microenvironment. Front Immunol. (2017) 8:248. 10.3389/fimmu.2017.00248 28337200 PMC5340776

[8] Dunn GP Bruce AT Ikeda H Old LJ Schreiber RD. Cancer immunoediting: from immunosurveillance to tumor escape. Nat Immunol. (2002) 3:991–8. 10.1038/ni1102-991 12407406

[9] Gajewski TF Meng Y Harlin H. Immune suppression in the tumor microenvironment. J Immunother. (2006) 29:233–40. 10.1097/01.cji.0000199193.29048.56 16699366

[10] Wu AA Drake V Huang HS Chiu S Zheng L. Reprogramming the tumor microenvironment: tumor-induced immunosuppressive factors paralyze T cells. Oncoimmunology (2015) 4:e1016700. 10.1080/2162402X.2015.1016700 26140242 PMC4485788

[11] Marcucci F Rumio C Corti A. Tumor cell-associated immune checkpoint molecules - Drivers of malignancy and stemness. Biochim Biophys Acta (2017) 1868:571–83. 10.1016/j.bbcan.2017.10.006 29056539

[12] Dong P Xiong Y Yue J Hanley SJB Watari H. B7H3 As a Promoter of metastasis and promising therapeutic target. Front Oncol. (2018) 8:264. 10.3389/fonc.2018.00264 30035102 PMC6043641

[13] Alsaab HO Sau S Alzhrani R Tatiparti K Bhise K Kashaw SK. PD-1 and PD-L1 checkpoint signaling inhibition for cancer immunotherapy: mechanism, combinations, and clinical outcome. Front Pharmacol. (2017) 8:561. 10.3389/fphar.2017.00561 28878676 PMC5572324

[14] Ostrand-Rosenberg S Horn LA Haile ST. The programmed death-1 immune-suppressive pathway: barrier to antitumor immunity. J Immunol. (2014) 193:3835–41. 10.4049/jimmunol.1401572 25281753 PMC4185425

[15] Gibbons Johnson RM Dong H Functional Expression of Programmed Death-Ligand 1 (B7-H1) by immune cells and tumor cells. Front Immunol. (2017) 8:961 10.3389/fimmu.2017.00961 28848559 PMC5554355

[16] Zhao LW Li C Zhang RL Xue HG Zhang FX Zhang F. B7-H1 and B7-H4 expression in colorectal carcinoma: correlation with tumor FOXP3(+) regulatory T-cell infiltration. Acta Histochem. (2014) 116:1163–8. 10.1016/j.acthis.2014.06.003 25053455

[17] Mu CY Huang JA Chen Y Chen C Zhang XG. High expression of PD-L1 in lung cancer may contribute to poor prognosis and tumor cells immune escape through suppressing tumor infiltrating dendritic cells maturation. Med Oncol. (2011) 28:682–8. 10.1007/s12032-010-9515-2 20373055

[18] Wang L Ma Q Chen X Guo K Li J Zhang M. Clinical significance of B7-H1 and B7-1 expressions in pancreatic carcinoma. World J Surg. (2010) 34:1059–65. 10.1007/s00268-010-0448-x 20145927

[19] Gao Q Wang XY Qiu SJ Yamato I Sho M Nakajima Y. Overexpression of PD-L1 significantly associates with tumor aggressiveness and postoperative recurrence in human hepatocellular carcinoma. Clin Cancer Res. (2009) 15:971–9. 10.1158/1078-0432.CCR-08-1608 19188168

[20] Böger C Behrens HM Mathiak M Krüger S Kalthoff H Röcken C. PD-L1 is an independent prognostic predictor in gastric cancer of Western patients. Oncotarget (2016) 7:24269–83. 10.18632/oncotarget.8169 27009855 PMC5029700

[21] Hamanishi J Mandai M Iwasaki M Okazaki T Tanaka Y Yamaguchi K. Programmed cell death 1 ligand 1 and tumor-infiltrating CD8^+^ T lymphocytes are prognostic factors of human ovarian cancer. Proc Natl Acad Sci USA. (2007) 104:3360–5. 10.1073/pnas.0611533104 17360651 PMC1805580

[22] Liu J Liu Y Wang W Wang C Che Y. Expression of immune checkpoint molecules in endometrial carcinoma. Exp Ther Med. (2015) 10:1947–52. 10.3892/etm.2015.2714 26640578 PMC4665362

[23] Kharma B Baba T Matsumura N Kang HS Hamanishi J Murakami R TAT1 drives tumor progression in serous papillary endometrial cancer. Cancer Res. (2014) 74:6519–30. 10.1158/0008-5472.CAN-14-0847 25267067

[24] Mezache L Paniccia B Nyinawabera A Nuovo GJ. Enhanced expression of PD L1 in cervical intraepithelial neoplasia and cervical cancers. Mod Pathol. (2015) 28:1594–602. 10.1038/modpathol.2015.108 26403783

[25] Dong P Xiong Y Yu J Chen L Tao T Yi S. Control of PD-L1 expression by miR-140/142/340/383 and oncogenic activation of the OCT4-miR-18a pathway in cervical cancer. Oncogene (2018) 10.1038/s41388-018-0347-4 29855617 PMC6160397

[26] Cao L Wang X Li S Zhi Q Wang Y Wang L. PD-L1 is a Prognostic Biomarker in Resected NSCLC patients with moderate/high smoking history and elevated serum SCCA level. J Cancer (2017) 8:3251–60. 10.7150/jca.21118 29158797 PMC5665041

[27] Saito H Kono Y Murakami Y Shishido Y Kuroda H Matsunaga T. Highly activated PD-1/PD-L1 pathway in gastric cancer with PD-L1 expression. Anticancer Res. (2018) 38:107–12. 10.21873/anticanres.12197 29277762

[28] Yagi T Baba Y Ishimoto T Iwatsuki M Miyamoto Y Yoshida N. PD-L1 expression, tumor-infiltrating lymphocytes, and clinical outcome in patients with surgically resected esophageal cancer. Ann Surg. (2017). 10.1097/SLA.0000000000002616. [Epub ahead of print]. 29206673

[29] Xue S Song G Yu J. The prognostic significance of PD-L1 expression in patients with glioma: A meta-analysis. Sci Rep. (2017) 7:4231. 10.1038/s41598-017-04023-x 28652622 PMC5484664

[30] Zhu J Wen H Bi R Wu Y Wu X. Prognostic value of programmed death-ligand 1 (PD-L1) expression in ovarian clear cell carcinoma. J Gynecol Oncol. (2017) 28:e77. 10.3802/jgo.2017.28.e77 29027395 PMC5641527

[31] Pyo JS Kang G Kim JY. Prognostic role of PD-L1 in malignant solid tumors: a meta-analysis. Int J Biol Markers (2017) 32:e68–74. 10.5301/jbm.5000225 27470134

[32] Wang X Teng F Kong L Yu J. PD-L1 expression in human cancers and its association with clinical outcomes. Onco Targets Ther. (2016) 9:5023–39. 10.2147/OTT.S105862 27574444 PMC4990391

[33] Lipson EJ Vincent JG Loyo M Kagohara LT Luber BS Wang H. PD-L1 expression in the Merkel cell carcinoma microenvironment: association with inflammation, Merkel cell polyomavirus and overall survival. Cancer Immunol Res. (2013) 1:54–63. 10.1158/2326-6066.CIR-13-0034 24416729 PMC3885978

[34] Heeren AM Punt S Bleeker MC Gaarenstroom KN van der Velden J Kenter GG. Prognostic effect of different PD-L1 expression patterns in squamous cell carcinoma and adenocarcinoma of the cervix. Mod Pathol. (2016) 29:753–63. 10.1038/modpathol.2016.64 27056074 PMC4931542

[35] George J Saito M Tsuta K Iwakawa R Shiraishi K Scheel AH. Genomic amplification of CD274 (PD-L1) in small-cell lung cancer. Clin Cancer Res. (2017) 23:1220–6. 10.1158/1078-0432.CCR-16-1069 27620277 PMC6329376

[36] Straub M Drecoll E Pfarr N Weichert W Langer R Hapfelmeier A. CD274/PD-L1 gene amplification and PD-L1 protein expression are common events in squamous cell carcinoma of the oral cavity. Oncotarget (2016) 7:12024–34. 10.18632/oncotarget.7593 26918453 PMC4914266

[37] Howitt BE Sun HH Roemer MG Kelley A Chapuy B Aviki E. Genetic basis for PD-L1 expression in squamous cell carcinomas of the cervix and vulva. JAMA Oncol. (2016) 2:518–22. 10.1001/jamaoncol.2015.6326 26913631

[38] Budczies J Denkert C Gyorffy B Schirmacher P Stenzinger A. Chromosome 9p copy number gains involving PD-L1 are associated with a specific proliferation and immune-modulating gene expression program active across major cancer types. BMC Med Genomics (2017) 10:74. 10.1186/s12920-017-0308-8 29212506 PMC5719741

[39] Barrett MT Anderson KS Lenkiewicz E Andreozzi M Cunliffe HE Klassen CL. Genomic amplification of 9p24.1 targeting JAK2, PD-L1, and PD-L2 is enriched in high-risk triple negative breast cancer. Oncotarget (2015) 6:26483–93. 10.18632/oncotarget.4494 26317899 PMC4694916

[40] Cancer Genome Atlas Research Network Comprehensive molecular characterization of gastric adenocarcinoma. Nature (2014) 513:202–9. 10.1038/nature13480 25079317 PMC4170219

[41] Cerami E Gao J Dogrusoz U Gross BE Sumer SO Aksoy BA. The cBio cancer genomics portal: an open platform for exploring multidimensional cancer genomics data. Cancer Discov. (2012) 2:401–4. 10.1158/2159-8290.CD-12-0095 22588877 PMC3956037

[42] Shin G Kang TW Yang S Baek SJ Jeong YS Kim SY. GENT: gene expression database of normal and tumor tissues. Cancer Inform. (2011) 10:149–57. 10.4137/CIN.S7226 21695066 PMC3118449

[43] Huang WY Hsu SD Huang HY Sun YM Chou CH Weng SL. MethHC: a database of DNA methylation and gene expression in human cancer. Nucleic Acids Res. (2015) 43:D856–61. 10.1093/nar/gku1151 25398901 PMC4383953

[44] Kataoka K Shiraishi Y Takeda Y Sakata S Matsumoto M Nagano S. Aberrant PD-L1 expression through 3'-UTR disruption in multiple cancers. Nature (2016) 534:402–6. 10.1038/nature18294 27281199

[45] Chen J Jiang CC Jin L Zhang XD. Regulation of PD-L1: a novel role of pro-survival signalling in cancer. Ann Oncol. (2016) 27:409–16. 10.1093/annonc/mdv615 26681673

[46] Mamessier E Birnbaum DJ Finetti P Birnbaum D Bertucci F. CMTM6 stabilizes PD-L1 expression and refines its prognostic value in tumors. Ann Transl Med. (2018) 6:54. 10.21037/atm.2017.11.26 29610746 PMC5879522

[47] Okita R Maeda A Shimizu K Nojima Y Saisho S Nakata M. PD-L1 overexpression is partially regulated by EGFR/HER2 signaling and associated with poor prognosis in patients with non-small-cell lung cancer. Cancer Immunol Immunother. (2017) 66:865–76. 10.1007/s00262-017-1986-y 28341875 PMC11028751

[48] Chen N Fang W Lin Z Peng P Wang J Zhan J. KRAS mutation-induced upregulation of PD-L1 mediates immune escape in human lung adenocarcinoma. Cancer Immunol Immunother. (2017) 66:1175–87. 10.1007/s00262-017-2005-z 28451792 PMC5579171

[49] Coelho MA de Carné Trécesson S Rana S Zecchin D Moore C Molina-Arcas M. Oncogenic RAS signaling promotes tumor immunoresistance by stabilizing PD-L1 mRNA. Immunity (2017) 47:1083–99.e6. 10.1016/j.immuni.2017.11.016 29246442 PMC5746170

[50] Crane CA Panner A Murray JC Wilson SP Xu H Chen L. PI(3) kinase is associated with a mechanism of immunoresistance in breast and prostate cancer. Oncogene (2009) 28:306–12. 10.1038/onc.2008.384 18850006 PMC3786571

[51] Parsa AT Waldron JS Panner A Crane CA Parney IF Barry JJ. Loss of tumor suppressor PTEN function increases B7-H1 expression and immunoresistance in glioma. Nat Med. (2007) 13:84–8. 10.1038/nm1517 17159987

[52] Deng Y Wang F Hughes T Yu J. FOXOs in cancer immunity: Knowns and unknowns. Semin Cancer Biol. (2018) 50:53–64. 10.1016/j.semcancer.2018.01.005 29309928 PMC5986596

[53] Yang W Lu YP Yang YZ Kang JR Jin YD Wang HW. Expressions of programmed death (PD)-1 and PD-1 ligand (PD-L1) in cervical intraepithelial neoplasia and cervical squamous cell carcinomas are of prognostic value and associated with human papillomavirus status. J Obstet Gynaecol Res. (2017) 43:1602–12. 10.1111/jog.13411 28833798

[54] Maeda T Hiraki M Jin C Rajabi H Tagde A Alam M. MUC1-C Induces PD-L1 and immune evasion in triple-negative breast cancer. Cancer Res. (2018) 78:205–15. 10.1158/0008-5472.CAN-17-1636 29263152 PMC5754244

[55] Pyzer AR Stroopinsky D Rosenblatt J Anastasiadou E Rajabi H Washington A. MUC1 inhibition leads to decrease in PD-L1 levels via upregulation of miRNAs. Leukemia (2017) 31:2780–90. 10.1038/leu.2017.163 28555079 PMC5791150

[56] Cortez MA Ivan C Valdecanas D Wang X Peltier HJ Ye Y. PDL1 Regulation by p53 via miR-34. J Natl Cancer Inst. (2015) 108:djv303. 10.1093/jnci/djv303 26577528 PMC4862407

[57] Chang CJ Chao CH Xia W Yang JY Xiong Y Li CW. p53 regulates epithelial-mesenchymal transition and stem cell properties through modulating miRNAs. Nat Cell Biol. (2011) 13:317–23. 10.1038/ncb2173 21336307 PMC3075845

[58] Wang YD Cai N Wu XL Cao HZ Xie LL Zheng PS. OCT4 promotes tumorigenesis and inhibits apoptosis of cervical cancer cells by miR-125b/BAK1 pathway. Cell Death Dis. (2013) 4:e760. 10.1038/cddis.2013.272 23928699 PMC3763434

[59] Zhong F Cheng X Sun S Zhou J. Transcriptional activation of PD-L1 by Sox2 contributes to the proliferation of hepatocellular carcinoma cells. Oncol Rep. (2017) 37:3061–7. 10.3892/or.2017.5523 28339084

[60] Janse van Rensburg HJ Azad T Ling M Hao Y Snetsinger B Khanal P The hippo pathway component TAZ promotes immune evasion in human cancer through PD-L1. Cancer Res. (2018) 78:1457–70. 10.1158/0008-5472.CAN-17-3139 29339539

[61] Bill R Christofori G. The relevance of EMT in breast cancer metastasis: Correlation or causality? FEBS Lett (2015) 589:1577–87. 10.1016/j.febslet.2015.05.002 25979173

[62] Kim S Koh J Kim MY Kwon D Go H Kim YA. PD-L1 expression is associated with epithelial-to-mesenchymal transition in adenocarcinoma of the lung. Hum Pathol. (2016) 58:7–14. 10.1016/j.humpath.2016.07.007 27473266

[63] Tsutsumi S Saeki H Nakashima Y Ito S Oki E Morita M. Programmed death-ligand 1 expression at tumor invasive front is associated with epithelial-mesenchymal transition and poor prognosis in esophageal squamous cell carcinoma. Cancer Sci. (2017) 108:1119–27. 10.1111/cas.13237 28294486 PMC5480087

[64] Guo M Tomoshige K Meister M Muley T Fukazawa T Tsuchiya T. Gene signature driving invasive mucinous adenocarcinoma of the lung. EMBO Mol Med. (2017) 9:462–81. 10.15252/emmm.201606711 28255028 PMC5376761

[65] Wang S Xu L Che X Li C Xu L Hou K. E3 ubiquitin ligases Cbl-b and c-Cbl downregulate PD-L1 in EGFR wild-type non-small cell lung cancer. FEBS Lett. (2018) 592:621–30. 10.1002/1873-3468.12985 29364514

[66] Cao X Zhao Y Wang J Dai B Gentile E Lin J. TUSC2 downregulates PD-L1 expression in non-small cell lung cancer (NSCLC). Oncotarget (2017) 8:107621–9. 10.18632/oncotarget.22581 29296193 PMC5746095

[67] Zhang J Bu X Wang H Zhu Y Geng Y Nihira NT. Cyclin D-CDK4 kinase destabilizes PD-L1 via cullin 3-SPOP to control cancer immune surveillance. Nature (2018) 553:91–5. 10.1038/nature25015 29160310 PMC5754234

[68] Dong P Xiong Y Watari H Hanley SJ Konno Y Ihira K. Suppression of iASPP-dependent aggressiveness in cervical cancer through reversal of methylation silencing of microRNA-124. Sci Rep. (2016) 6:35480. 10.1038/srep35480 27765948 PMC5073231

[69] Ihira K Dong P Xiong Y Watari H Konno Y Hanley SJ. EZH2 inhibition suppresses endometrial cancer progression via miR-361/Twist axis. Oncotarget (2017) 8:13509–20. 10.18632/oncotarget.14586 28088786 PMC5355116

[70] Dong P Ihira K Xiong Y Watari H Hanley SJ Yamada T. Reactivation of epigenetically silenced miR-124 reverses the epithelial-to-mesenchymal transition and inhibits invasion in endometrial cancer cells via the direct repression of IQGAP1 expression. Oncotarget (2016) 7:20260–70. 10.18632/oncotarget.7754 26934121 PMC4991452

[71] Zhang Y Xiang C Wang Y Duan Y Liu C Zhang Y. PD-L1 promoter methylation mediates the resistance response to anti-PD-1 therapy in NSCLC patients with EGFR-TKI resistance. Oncotarget (2017) 8:101535–44. 10.18632/oncotarget.21328 29254184 PMC5731894

[72] Wang YF Liu F Sherwin S Farrelly M Yan XG Croft A. Cooperativity of HOXA5 and STAT3 is critical for HDAC8 inhibition-mediated transcriptional activation of PD-L1 in human melanoma cells. J Invest Dermatol. (2018) 138:922–32. 10.1016/j.jid.2017.11.009 29174371

[73] Kao SC Cheng YY Williams M Kirschner MB Madore J Lum T. Tumor suppressor microRNAs contribute to the regulation of PD-L1 expression in malignant pleural mesothelioma. J Thorac Oncol. (2017) 12:1421–33. 10.1016/j.jtho.2017.05.024 28629895

[74] Audrito V Serra S Stingi A Orso F Gaudino F Bologna C. PD-L1 up-regulation in melanoma increases disease aggressiveness and is mediated through miR-17-5p. Oncotarget (2017) 8:15894–911. 10.18632/oncotarget.15213 28199980 PMC5362532

[75] Cioffi M Trabulo SM Vallespinos M Raj D Kheir TB Lin ML. The miR-25-93-106b cluster regulates tumor metastasis and immune evasion via modulation of CXCL12 and PD-L1. Oncotarget (2017) 8:21609–25. 10.18632/oncotarget.15450 28423491 PMC5400610

[76] Zhao L Yu H Yi S Peng X Su P Xiao Z. The tumor suppressor miR-138-5p targets PD-L1 in colorectal cancer. Oncotarget (2016) 7:45370–84. 10.18632/oncotarget.9659 27248318 PMC5216728

[77] Wang Y Wang D Xie G Yin Y Zhao E Tao K. MicroRNA-152 regulates immune response via targeting B7-H1 in gastric carcinoma. Oncotarget (2017) 8:28125–34. 10.18632/oncotarget.15924 28427226 PMC5438636

[78] Yee D Shah KM Coles MC Sharp TV Lagos D. MicroRNA-155 induction via TNF-α and IFN-γ suppresses expression of programmed death ligand-1 (PD-L1) in human primary cells. J Biol Chem. (2017) 292:20683–93. 10.1074/jbc.M117.809053 29066622 PMC5733604

[79] Holla S Stephen-Victor E Prakhar P Sharma M Saha C Udupa V. Mycobacteria-responsive sonic hedgehog signaling mediates programmed death-ligand 1- and prostaglandin E2-induced regulatory T cell expansion. Sci Rep. (2016) 6:24193. 10.1038/srep24193 27080341 PMC4832185

[80] Xu S Tao Z Hai B Liang H Shi Y Wang T. miR-424(322) reverses chemoresistance via T-cell immune response activation by blocking the PD-L1 immune checkpoint. Nat Commun. (2016) 7:11406. 10.1038/ncomms11406 27147225 PMC4858750

[81] Fujita Y Yagishita S Hagiwara K Yoshioka Y Kosaka N Takeshita F. The clinical relevance of the miR-197/CKS1B/STAT3-mediated PD-L1 network in chemoresistant non-small-cell lung cancer. Mol Ther. (2015) 23:717–27. 10.1038/mt.2015.10 25597412 PMC4395779

[82] Zhu J Chen L Zou L Yang P Wu R Mao Y. MiR-20b,−21, and−130b inhibit PTEN expression resulting in B7-H1 over-expression in advanced colorectal cancer. Hum Immunol. (2014) 75:348–53. 10.1016/j.humimm.2014.01.006 24468585

[83] Garcia-Diaz A Shin DS Moreno BH Saco J Escuin-Ordinas H Rodriguez GA. Interferon receptor signaling pathways regulating PD-L1 and PD-L2 expression. Cell Rep. (2017) 19:1189–201. 10.1016/j.celrep.2017.04.031 28494868 PMC6420824

[84] Wang X Yang L Huang F Zhang Q Liu S Ma L. Inflammatory cytokines IL-17 and TNF-α up-regulate PD-L1 expression in human prostate and colon cancer cells. Immunol Lett. (2017) 184:7–14. 10.1016/j.imlet.2017.02.006 28223102 PMC5362328

[85] Alsuliman A Colak D Al-Harazi O Fitwi H Tulbah A Al-Tweigeri T. Bidirectional crosstalk between PD-L1 expression and epithelial to mesenchymal transition: significance in claudin-low breast cancer cells. Mol Cancer (2015) 14:149. 10.1186/s12943-015-0421-2 26245467 PMC4527106

[86] Noman MZ Desantis G Janji B Hasmim M Karray S Dessen P. PD-L1 is a novel direct target of HIF-1α, and its blockade under hypoxia enhanced MDSC-mediated T cell activation. J Exp Med. (2014) 211:781–90. 10.1084/jem.20131916 24778419 PMC4010891

[87] Liu C Lu J Tian H Du W Zhao L Feng J Increased expression of PD-L1 by the human papillomavirus 16 E7 oncoprotein inhibits anticancer immunity. Mol Med Rep. (2017) 15:1063–70. 10.3892/mmr.2017.6102 28075442 PMC5367331

[88] Yang L Huang F Mei J Wang X Zhang Q Wang H. Posttranscriptional control of PD-L1 expression by 17β-estradiol via PI3K/Akt signaling pathway in ERα-Positive Cancer Cell Lines. Int J Gynecol Cancer (2017) 27:196–205. 10.1097/IGC.0000000000000875 27870715 PMC5258765

[89] Li J Chen L Xiong Y Zheng X Xie Q Zhou Q. Knockdown of PD-L1 in human gastric cancer cells inhibits tumor progression and improves the cytotoxic sensitivity to CIK therapy. Cell Physiol Biochem. (2017) 41:907–20. 10.1159/000460504 28222426

[90] Liao Y Chen L Feng Y Shen J Gao Y Cote G. Targeting programmed cell death ligand 1 by CRISPR/Cas9 in osteosarcoma cells. Oncotarget (2017) 8:30276–87. 10.18632/oncotarget.16326 28415820 PMC5444742

[91] Chen L Xiong Y Li J Zheng X Zhou Q Turner A. PD-L1 expression promotes epithelial to mesenchymal transition in human esophageal cancer. Cell Physiol Biochem. (2017) 42:2267–80. 10.1159/000480000 28848143

[92] Li Y Yang X Wu Y Zhao K Ye Z Zhu J. B7-H3 promotes gastric cancer cell migration and invasion. Oncotarget (2017) 8:71725–35. 10.18632/oncotarget.17847 29069741 PMC5641084

[93] Jinesh GG Manyam GC Mmeje CO Baggerly KA Kamat AM. Surface PD-L1, E-cadherin, CD24, and VEGFR2 as markers of epithelial cancer stem cells associated with rapid tumorigenesis. Sci Rep. (2017) 7:9602. 10.1038/s41598-017-08796-z 28851898 PMC5575243

[94] Nishino M Ozaki M Hegab AE Hamamoto J Kagawa S Arai D. Variant CD44 expression is enriching for a cell population with cancer stem cell-like characteristics in human lung adenocarcinoma. J Cancer (2017) 8:1774–85. 10.7150/jca.19732 28819374 PMC5556640

[95] Lee Y Shin JH Longmire M Wang H Kohrt HE Chang HY. CD44^+^ cells in head and neck squamous cell carcinoma suppress T-cell-mediated immunity by selective constitutive and inducible expression of PD-L1. Clin Cancer Res. (2016) 22:3571–81. 10.1158/1078-0432.CCR-15-2665 26864211 PMC5623594

[96] Zhi Y Mou Z Chen J He Y Dong H Fu X. B7H1 expression and epithelial-to-mesenchymal transition phenotypes on colorectal cancer stem-like cells. PLoS ONE (2015) 10:e0135528. 10.1371/journal.pone.0135528 26284927 PMC4540313

[97] Gupta HB Clark CA Yuan B Sareddy G Pandeswara S Padron AS. Tumor cell-intrinsic PD-L1 promotes tumor-initiating cell generation and functions in melanoma and ovarian cancer. Signal Transduct Target Ther (2016) 1:16030. 10.1038/sigtrans.2016.30 28798885 PMC5547561

[98] Almozyan S Colak D Mansour F Alaiya A Al-Harazi O Qattan A. PD-L1 promotes OCT4 and Nanog expression in breast cancer stem cells by sustaining PI3K/AKT pathway activation. Int J Cancer (2017) 141:1402–12. 10.1002/ijc.30834 28614911 PMC5575465

[99] Qiu XY Hu DX Chen WQ Chen RQ Qian SR Li CY. PD-L1 confers glioblastoma multiforme malignancy via Ras binding and Ras/Erk/EMT activation. Biochim Biophys Acta (2018) 1864(5 Pt A):1754–69. 10.1016/j.bbadis.2018.03.002 29510196

[100] Wang Y Wang H Zhao Q Xia Y Hu X Guo J. PD-L1 induces epithelial-to-mesenchymal transition via activating SREBP-1c in renal cell carcinoma. Med Oncol. (2015) 32:212. 10.1007/s12032-015-0655-2 26141060

[101] Liu S Chen S Yuan W Wang H Chen K Li D. PD-1/PD-L1 interaction up-regulates MDR1/P-gp expression in breast cancer cells via PI3K/AKT and MAPK/ERK pathways. Oncotarget (2017) 8:99901–12. 10.18632/oncotarget.21914 29245948 PMC5725139

[102] Tamai K Nakamura M Mizuma M Mochizuki M Yokoyama M Endo H. Suppressive expression of CD274 increases tumorigenesis and cancer stem cell phenotypes in cholangiocarcinoma. Cancer Sci. (2014) 105:667–74. 10.1111/cas.12406 24673799 PMC4317902

